# Artificial insemination and parthenogenesis in the whitespotted bamboo shark *Chiloscyllium plagiosum*

**DOI:** 10.1038/s41598-021-88568-y

**Published:** 2021-05-13

**Authors:** Jennifer T. Wyffels, Lance M. Adams, Frank Bulman, Ari Fustukjian, Michael W. Hyatt, Kevin A. Feldheim, Linda M. Penfold

**Affiliations:** 1 South-East Zoo Alliance for Reproduction & Conservation, Yulee, FL 32097 USA; 2 Aquarium of the Pacific, 100 Aquarium Way, Long Beach, CA 90802 USA; 3 Ripley’s Aquarium of the Smokies, 88 River Road, Gatlinburg, TN 37738 USA; 4 The Florida Aquarium, 701 Channelside Drive, Tampa, FL 33602 USA; 5 Adventure Aquarium, 1 Riverside Drive, Camden, NJ 08103 USA; 6 Pritzker Laboratory for Molecular Systematics and Evolution, Field Museum of Natural History, 1400 S. Lake Shore Drive, Chicago, IL 60605 USA; 7 Present Address: University of Delaware, Center for Bioinformatics & Computational Biology, 590 Avenue 1743, Newark, DE 19713 USA; 8 Present Address: Loveland Living Planet Aquarium, 12033 Lone Peak Pkwy, Draper, UT 84020 USA; 9 Present Address: New York Aquarium, Wildlife Conservation Society, 602 Surf Ave., Brooklyn, NY 11224 USA

**Keywords:** Reproductive biology, Ichthyology, Animal physiology

## Abstract

Non-lethal methods for semen collection from elasmobranchs to better understand species reproduction has accompanied the development of artificial insemination. Ejaculates (n = 82) collected from whitespotted bamboo sharks *Chiloscyllium plagiosum* (n = 19) were assessed and cold-stored raw or extended at 4 °C. Females (n = 20) were inseminated with fresh or 24–48 h cold-stored raw or extended semen and paternity of offspring determined with microsatellite markers. Insemination of females with fresh semen (n = 10) resulted in 80 hatchlings and 27.6% fertility. Insemination of females with semen cold-stored 24 h (n = 4) and 48 h (n = 1) semen resulted in 17 hatchlings and fertilization rates of 28.1% and 7.1% respectively. Two females inseminated with fresh or cold-stored semen laid eggs that hatched from fertilization and parthenogenesis within the same clutch. Parthenogenesis rate for inseminated females was 0.71%. Results demonstrate artificial insemination with cold-stored semen can provide a strategy for transport of male genetics nationally and internationally, precluding the need to transport sharks. Production of parthenotes in the same clutch as sexually fertilized eggs highlights the prevalence of parthenogenesis in whitespotted bamboo sharks and poses important considerations for population management.

## Introduction

Whitespotted bamboo sharks *Chiloscyllium plagiosum* are endemic to the Indo-West Pacific Ocean and common in aquariums worldwide because of their longevity, successful reproduction under managed care and small size. They are classified as “Near Threatened” by the International Union for Conservation of Nature (IUCN) Red List^1,2^ due to habitat destruction and unmanaged fisheries activity. Whitespotted bamboo sharks are oviparous and reproduce readily in aquaria laying eggs in pairs, every 6–7 days during a 2–3 month annual laying cycle^3–6^.

Whitespotted bamboo females do not require frequent and repeated mating during their laying season and instead use sperm stored in the nidamental (shell or oviducal) gland to fertilize eggs^3,4^. Whitespotted bamboo sharks also have been shown to reproduce parthenogenetically, adding another level of complexity to managing reproduction^7,8^. To facilitate reproduction and maintain genetic diversity, assisted reproductive technologies including artificial insemination (AI) are one solution^9,10^ that avoids the extra challenges, risks and costs associated with transporting sharks between institutions. Artificial insemination among elasmobranchs with the production of offspring has been accomplished for four shark species, including this species, (cloudy catshark *Scyliorhinus torazame*^11^, whitespotted bamboo shark *C. plagiosum*^12^, brownbanded bamboo shark *Chiloscyllium punctatum*^9^, zebra or leopard shark *Stegostoma tigrinum*^9^) and one skate species, (clearnose skate *Rostroraja eglanteria*^13^) using freshly collected, but not cold-stored semen.

The first steps for developing AI for many species include semen collection and characterization. Semen for analysis has historically been collected from elasmobranchs following euthanasia^14–21^ but more recently, protocols for non-lethal sampling have been developed^22^. Cold storage (1–4 °C) is an easy and convenient method for short-term preservation of fish sperm and eggs for hours to weeks with the conditions of storage such as antibiotics and diluents optimized for each species^23–28^. Gamete cold storage does not require specialized equipment and could be useful in aquariums to allow the movement of genetic material between institutions to maintain gene diversity in a population without the need to transport an animal. This is especially useful as cryopreservation research using elasmobranch semen is limited to a few species of stingray^9,15^ and investigation into cryopreservation of semen from whitespotted bamboo sharks has not yielded viable sperm (Wyffels, unpublished).

The ability of elasmobranchs to reproduce by parthenogenesis is widely known for sharks (swell shark *Cephaloscyllium ventriosum*^29^; bonnethead shark *Sphyrna tiburo*^30^; blacktip shark *Carcharinus limbatus*^31^; whitespotted bamboo *C. plagiosum*^7^ and zebra sharks *S. tigrinum*^32,33^) and rays (spotted eagle ray *Aetobatus narinari*^34^), and documented for wild smalltooth sawfish *Prisitis pectinata*^35^. The strategy behind this form of reproduction is unknown, but it may be a reproductive adaptation for females after prolonged periods without male interaction. As such, it is assumed to occur instead of fertilization from a male. From a population management perspective, parthenotes do not contribute to the genetic diversity of a population and are rather female ‘clones’ of the dam. The overall incidence of parthenogenesis in whitespotted bamboo sharks is currently unknown, but realizing the incidence of parthenogenesis would aid with population modeling. Microsatellite markers have been developed previously to examine parentage in the whitespotted bamboo shark^7,43^.

The objectives of this study were to: (1) develop a safe and reliable method for non-lethal collection and characterization of semen from whitespotted bamboo sharks; (2) examine the effects of cold storage on sperm longevity; (3) produce offspring following AI with fresh and cold-stored semen; (4) develop additional microsatellite markers to confirm parentage of AI offspring; and (5) determine the incidence of parthenogenesis in whitespotted bamboo sharks.

## Results

### Ejaculate characteristics

**Figure 1:**
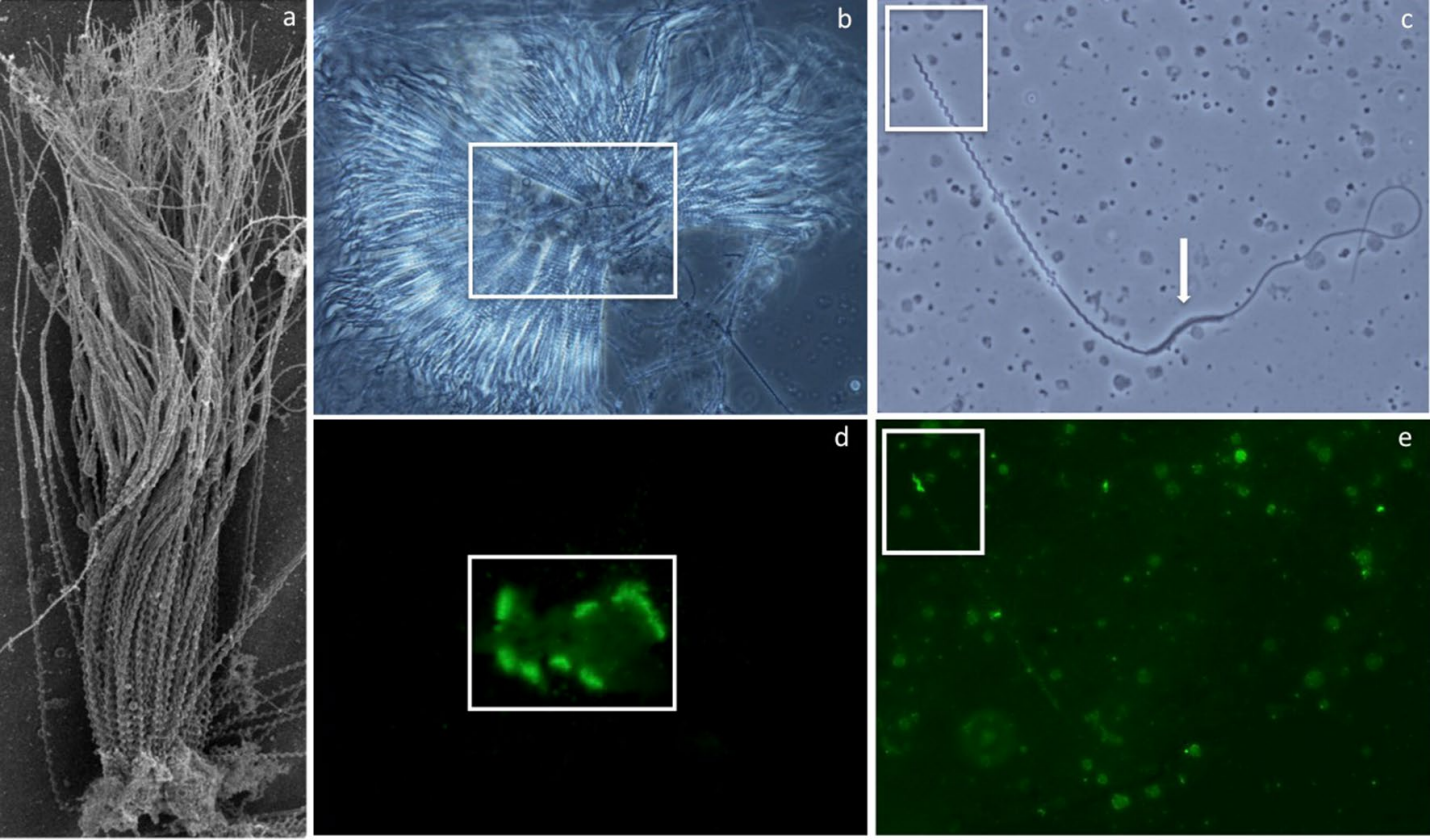
Whitespotted bamboo shark *Chiloscyllium plagiosum* spermatozuegmata and spermatozoa micrographs illustrating the morphology and acrosome location for aggregated and individual spermatozoa. Scanning electron micrograph of a spermatozeugma fragment (**a**) revealing sperm with heads closely aligned and embedded in a matrix core. Matching phase contrast and fluorescent micrographs of a spermatozeugma with radially aligned heads (**b**, **d**) and individual spermatozoan (**c**, **e**) with acrosomes highlighted by fluorescein isothiocyanate conjugated *Arachis hypogaea* agglutinin (PNA-FITC) stain. Whitespotted bamboo shark spermatozoa (**c**) possess a helical head with an acrosome (**e**), a helical midpiece and a flagellum with a transient cytoplasmic sleeve (arrow) at the junction of the midpiece and tail that was shed after acquiring motility.

Ejaculate collection was explored using two techniques (catheterization and manual expression) and collection by manual expression became the default collection method because of its relative simplicity and efficiency. A total of 82 ejaculates from 19 sharks were collected all months of the year attempted and semen characteristics are presented in Table 1. Ejaculates were light brown or buff colored, opaque and viscous containing individual sperm and spermatozeugmata composed of radially arrayed sperm with heads that were closely aligned and embedded in a matrix core (Fig. 1a)^21^. Spermatozeugmata were observed in 48% of ejaculates irrespective of time of year. Spermatozoa had elongated helical heads (Fig. 1c) with an apical acrosome that was stained by *Arachis hypogaea* lectin or peanut agglutinin (PNA) (Fig. 1b–e). Sperm morphology was 100% normal for all ejaculates. The length of the head, midpiece and flagellum as well as the number of gyres for spermatozoa are shown in Table 2.

**Table 1:** Whitespotted bamboo shark *Chiloscyllium plagiosum* semen characteristics.

	n*	mean ± SE	median	range
Volume (ml)	19 (82)	0.61 ± 0.08	0.52	0.05–2.3
Sperm concentration (10^6^/ml)	19 (73)	858 ± 129	799.5	3–4670
Spermatazeugmata (%)	19 (76)	23.51 ± 5.74	20	0–25
Plasma membrane integrity (%)	18 (80)	64 ± 5	71.05	49–95
Total motility (%)	16 (61)	65 ± 6	74.6	39–95
Progressive motility (%)	19 (79)	19 ± 4	17.5	1–70
pH	19 (78)	6.28 ± 0.04	6.28	5.5–7.5
Osmolarity (mOsm)	16 (60)	1091 ± 34	1115.86	682–1410

*Sharks (ejaculates).

**Table 2:** Whitespotted bamboo shark *Chiloscyllium plagiosum* spermatozoa morphometrics. Lengths from digital image analysis of 10 spermatozoa per shark from five sharks were measured and summarized as a mean and standard error (SE).

	Length (*µ*m) mean ± SE
Total	178.3 ± 0.8
Head	57.7 ± 0.2
Midpiece	19.9 ± 0.2
Flagellum	100.7 ± 0.6
Acrosome	3.0 ± 0.1
	**Gyres**
Total (Head & Midpiece)	34.8 ± 0.1
Head	25.9 ± 0.1

Spermatozoa in raw ejaculates had little or no motility but acquired motility after dilution in artificial seawater (ASW). After dilution in ASW, the cytoplasmic sleeve, found at the junction of the midpiece and tail (Fig. 1c), was observed to slide toward the distal end of the flagellum until it was shed from the spermatozoa. Shed cytoplasmic sleeves accumulated at the peripheral margin of the spermatozeugmata. Individual, free spermatozoa moving progressively had serpentine tails and demonstrated rotation about the long axis.

### Short-term semen storage

**Figure 2:**
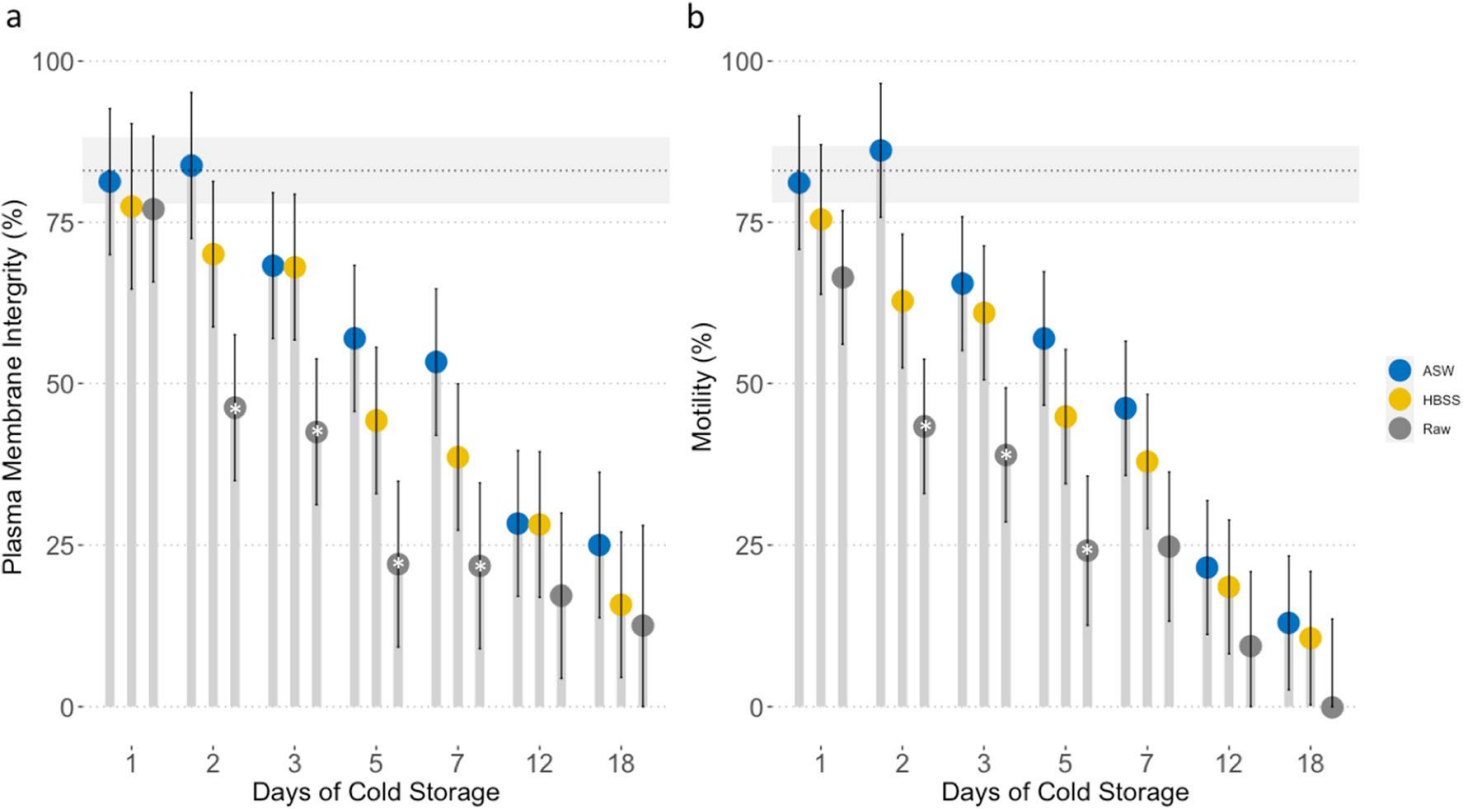
The effect of cold storage on whitespotted bamboo shark *Chiloscyllium plagiosum* ejaculates. Plasma membrane integrity (**a**) and motility (**b**) of ejaculates cold-stored raw or extended with elasmobranch modified Hank’s balanced salt solution (E-HBSS) or artificial seawater (ASW) and initial mean (dotted line) and standard error (grey rectangle) of ejaculates. Treatment bars are capped with a circle (estimated marginal mean) and error bars (95% confidence interval). *Indicates significant difference among treatments at each time point (*p* < 0.05).

Sample preparation method (*p* < 0.05) and duration of cold storage (*p* < 0.05) affected plasma membrane integrity (PMI) and total motility with diluted samples and shorter storage times resulting in higher PMI and total motility (Fig. 2). There was a strong positive correlation between sperm PMI and total motility (r = 0.93, n = 82, *p* < 0.001). There were no observed differences in PMI or total motility for semen extended in either ASW or elasmobranch adjusted Hank’s balanced salt solution (E-HBSS), both of which supported sperm motility and PMI for up to 18 days (Fig. 2). Semen stored raw retained PMI, however was completely immotile by day 18. Cold storage of raw ejaculate for 24 h did not affect PMI (*p* = 0.268) but total motility decreased (*p* = 0.009). For semen cold-stored for 2–7 days, ejaculates extended in ASW or E-HBSS had higher PMI and motility than samples stored raw (*p* < 0.05, Fig. 2). Samples stored raw declined in quality the fastest of the three preparation methods and had the lowest PMI and total motility each day samples were evaluated (Fig. 2).

### Artificial insemination and paternity

The time for insemination of each female took approximately 10 min and no adverse effects were observed. If egg cases were present in the oviduct the procedure was more challenging but the egg cases are leathery and durable and none were physically affected by the AI procedure, as evidenced by all egg cases laid being without damage. No eggs laid during the six weeks prior to AI developed. Fifteen females laid 114 fertile eggs resulting in 97 hatchlings after insemination and there was considerable variation in the number of eggs laid (1–43), duration or number of days of fertility (1–107), fertility (4.2–66.7%) and hatchability (2.6–60.0%) between females receiving 100–750 × 10^6^ spermatozoa in raw and extended inseminates.

**Figure 3:**
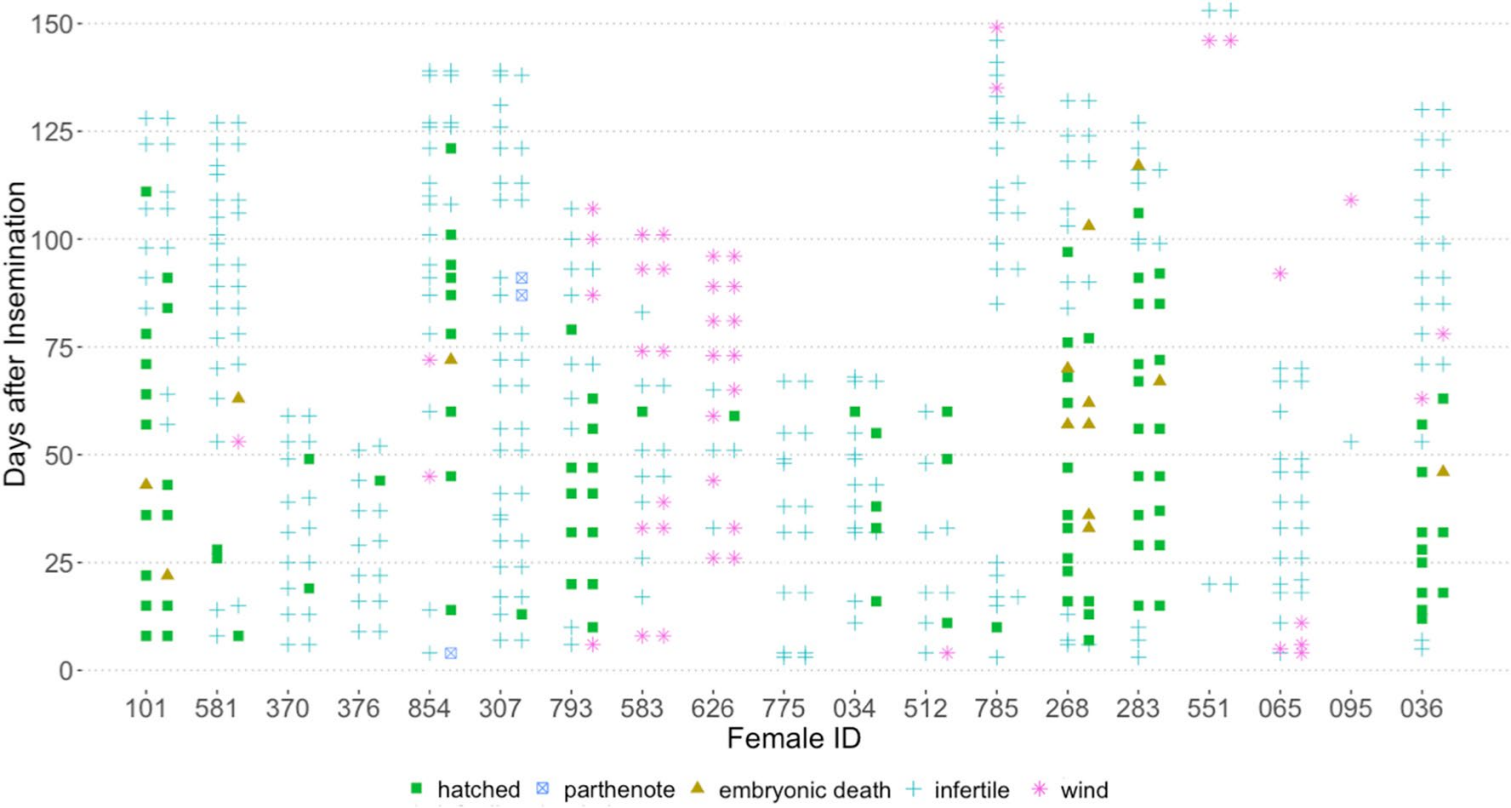
Oviposition dates for whitespotted bamboo shark *Chiloscyllium plagiosum* females after artificial insemination. Egg cases are usually laid in pairs, one egg from each side of the paired reproductive tract. Egg cases classified as fertile were assigned to categories: hatched, parthenote or embryonic death. Egg cases classified as infertile had ova without evidence of embryonic development. Empty or wind egg cases did not contain ova.

Five females laid eggs (n = 66) 1–153 days after AI but none were fertile (Table 3). For most females (n = 9), all fertile eggs completed development successfully, but three females laid one fertile egg, two females laid two fertile eggs, and one female laid seven fertile eggs that all died before hatching (Supplemental Table 1). Average (± standard deviation) incubation was 117 ± 10 days and ranged from 74–142 days (n = 73). Successful insemination of both oviducts was confirmed by observation of development of eggs laid as pairs and was observed for five females that incidentally also had the highest fertility (37.5–66.7%) and hatchability (34.4–60.0%) rates (Table 3). Bilateral insemination success was not associated with inseminate age (fresh or 24 h), preparation (raw or extended), or sperm dose (100–750 × 10^6^ total sperm) and therefore possibly related to the technique. For the remainder of the females in successful trials (n = 10), only one of a pair of egg cases laid together was fertile (Fig. 3).

**Table 3:** Outcome of whitespotted bamboo sharks *Chiloscyllium plagiosum* artificial insemination trials.

Female ID	Male ID-ejaculate ID	Egg cases laid	Fertility duration (days)	Days fertile	Hatchlings		Fertility (%)	Hatchability (%)
					AI	P		
854	320-E1	43	107	14–121	9	1	26.8%	22.0%
101	381-E2	30	103	8–111	15	–	**56.7%**	**50.0%**
283	381-E4	32	101	15–116	18	0	**66.7%**	**60.0%**
268	381-E5	36	97	6–103	14	0	**58.3%**	**38.9%**
793	395-E1^	27	69	10–79	12	0	**52.2%**	**52.2%**
036	320-E3	34	51	12–63	11	0	**37.5%**	**34.4%**
581	381-E1	32	55	8–63	3	0	12.9%	9.7%
512	381-E3	12	49	11–60	3	0	27.3%	27.3%
034	381-E3	19	44	16–60	5	0	26.3%	26.3%
370	2969-E1*	18	30	19–49	2	0	11.1%	11.1%
785	381-E4	26	1	10	1	0	4.2%	4.2%
376	381-E1	14	1	44	1	0	7.1%	7.1%
307	320-E1	39	1	13	1	2	7.7%	2.6%
626	395-E1^	19	1	59	1	0	20.0%	20.0%
583	395-E1^	22	1	60	1	0	9.1%	9.1%
063	381-E2	16	0	–	0	0	0%	0%
775	381-E3	16	0	–	0	0	0%	0%
551	381-E5	6	0	–	0	0	0%	0%
065	320-E2	26	0	–	0	0	0%	0%
095	320-E2	2	0	–	0	0	0%	0%

Hatchlings: developed from sexual fertilization (AI) or parthenogenetically (P) after artificial insemination. *Indicates semen shipped overnight from Adventure Aquarium, NJ, to Aquarium of the Pacific, CA, for artificial insemination. ^ Indicates semen shipped overnight from The Florida Aquarium, FL, to Ripley’s Aquarium of the Smokies, TN, for artificial insemination. Bold fertility and hatchability percentages indicate females where artificial insemination was confirmed bilaterally successful.

**Figure 4:**
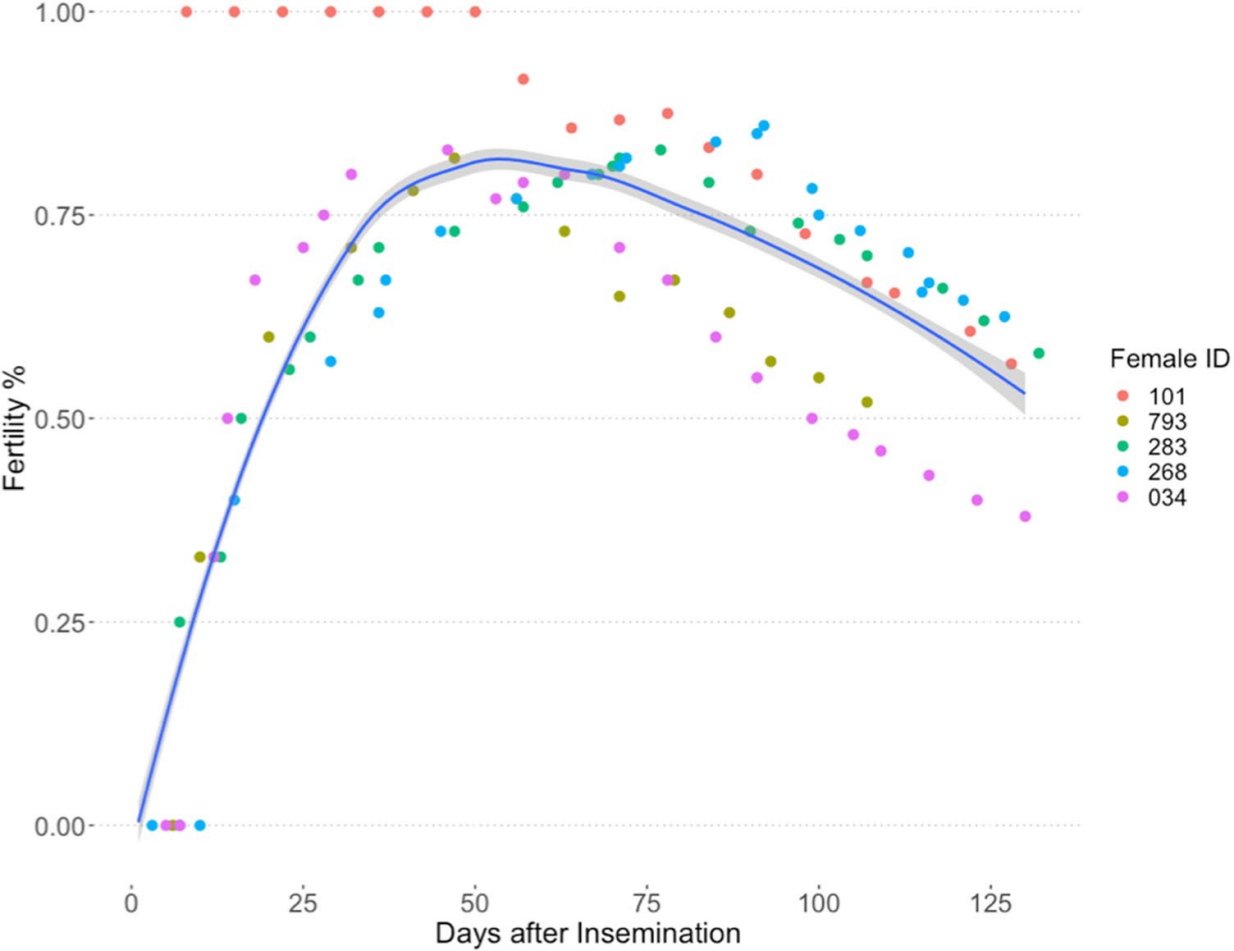
Fertility, expressed as a percentage of egg cases laid, for five females that stored sperm for more than 3 months from artificial insemination and laid pairs of fertile eggs. Fertility (smoothed loess local regression line) with 95% confidence interval (grey) for females (n = 5) beginning 6 days after and ending 116 days after artificial insemination. Individual female fertility is distinguished by marker color.

The combined average number of egg cases laid per females in successful trials was 24, double the combined average number of egg cases laid by females in unsuccessful trials, 12. The average fertility duration for females that produced nine or more hatchlings (n = 6) was 88 ± 9 days compared to 45 ± 5 days for females that produced 2–5 hatchlings (n = 4) (Table 3). All eggs laid for 43 days after AI were fertile for female (ID 101) and one egg hatched from the first pair of egg cases laid after AI by four females on day six, eight or 13 post-insemination (Fig. 3). Remaining females (n = 11) laid 1–10 infertile eggs before their first fertilized egg. Although females continued to lay fertile eggs for up to 121 days after insemination, fertility peaked by four weeks and decreased incrementally after 10 weeks (Fig. 4). All females that laid one or more fertile eggs also laid multiple infertile eggs afterwards, at the end of their laying cycle. Two females laid a single fertile egg 10 or 13 days post-insemination but not afterwards. Time from AI to laying date for the first fertilized egg ranged from 6–60 days and females laid fertile eggs 6–121 days after AI for 1–107 days (Table 3). There was no difference in fertility for ejaculates that were split and inseminated as raw semen or semen extended 1:1 with ASW (paired t-test: t = -0.08, df = 3, *p* = 0.9424). Collectively, nine females laid 61 fertilized eggs after AI using raw semen compared to 50 fertilized eggs laid by six females inseminated with extended semen (Supplemental Table 1).

Inseminates varied in sperm number from 3–750 × 10^6^ spermatozoa and fertile eggs resulted from all doses except the lowest dose, 3 × 10^6^ spermatozoa. However, only one of the two females that received the lowest dose laid eggs regularly post-insemination and can be considered in analyses. Insemination using 100–750 × 10^6^ spermatozoa was effective and there was high variability in the number of hatchlings produced among the females even when using semen from the same male. For example, among 10 females inseminated with fresh semen from male 381, seven females produced 1–18 hatchlings each and three females produced no hatchlings (Supplemental Table 1).

Cold-stored semen successfully fertilized eggs for up to 48 h after collection and inseminates from 24 h cold-stored semen had similar fertility and hatchability as inseminates from fresh semen. The same ejaculate split and used fresh and after 48 h of cold storage both fertilized eggs with three hatchlings from the fresh inseminate and one from the cold-stored inseminate. Two males contributed semen that was cold-stored for 24 h and shipped overnight between aquaria before being used for insemination, and both ejaculates fertilized eggs. Semen collected from male 395 at The Florida Aquarium and shipped overnight for AI trials at Ripley’s Aquarium of the Smokies was used to inseminate three females that resulted in a total of 14 hatchlings. Semen from Adventure Aquarium male 2969 was shipped to Aquarium of the Pacific and used to inseminate a female that laid two fertile eggs that successfully hatched. Combined, 17 hatchlings resulted from AI of five females with cold-stored semen from three males from three aquaria.

### Parthenogenesis

To determine the general incidence of parthenogenesis, 1053 eggs collected over 105 days from a habitat with 48 females were incubated and monitored for embryo development. Five eggs from 28 fertile eggs hatched. The hatchlings and nine of the 23 embryos (no DNA samples could be collected from 14) were genotyped and all hatchlings and seven of the nine embryos were parthenotes, which accounts for 1.1% of eggs laid. If the remaining embryos that could not be genotyped (n = 14) were all parthenotes, the maximum incidence of parthenogenesis in this population would be 2.5%. The minimum number of females responsible for parthenotes was four accounting for 8.3% of the 48 females, however some females in the population may not have been laying eggs, making the incidence higher (Table 4).

**Table 4:** Whitespotted bamboo shark *Chiloscyllium plagiosum* female and parthenote microsatellite loci.

Female ID	Egg ID*	Cp1	Cpl 80	Cpl 150	Cpl 471	Cpl 930	Cpl 1163	Cpl 962	Cpl 1161	Cpl 1141
**Female 1**		289/315	266/282	200/202	226/228	178/182	201/	356/	194/210	186/194
	P1	315/315	266/266	202/202	226/226	182/182	201/201	356/356	194/194	194/194
	P2	289/289	282/282	200/200	226/226	178/178	201/201	356/356	210/210	186/186
	P3	289/289	266/266	202/202	228/228	182/182	201/201	356/356	194/194	194/194
	P4	315/315	266/266	202/202	226/226	178/178	201/201	356/356	210/210	194/194
**Female 2**		289/	274/294	194/202	228/	176/182	193/	368/	200/210	194/
	P5	289/289	294/294	194/194	228/228	182/182	193/193	368/368	210/210	194/194
	P6	289/289	274/274	202/202	228/228	176/176	193/193	368/368	210/210	194/194
	P7	289/289	294/294	202/202	228/228	182/182	193/193	368/368	200/200	194/194
	P8	−/−	294/294	−/−	−/−	−/−	193/193	356/356	200/200	194/194
**Female 3**		289/289	266/282	194/−	228/−	176/182	201/−	364/−	198/210	186/194
	P9	289/289	282/282	194/194	228/228	182/182	201/201	−/−	210/210	186/186
	P10	289/289	266/266	194/194	228/228	176/176	201/201	364/364	198/198	194/194
	P11	289/289	266/266	194/194	228/228	182/182	201/201	364/364	210/210	194/194
**Female 4**		315/−	266/−	194/−	226/−	178/−	193/−	368/−	200/−	194/−
	P12	315/315	266/266	194/194	226/226	178/178	193/193	368/368	200/200	194/194
**Female 854**		315/315	266/286	194/194	226/228	176/178	193/193	304/368	198/200	186/194
	AI-P1	315/315	286/286	194/194	226/226	176/176	193/193	304/304	198/198	186/186
**Female 307**		315/315	274/274	194/194	226/228	182/182	193/201	360/364	200/208	186/194
	AI-P2	315/315	274/274	194/194	226/226	182/182	201/201	360/360	208/208	194/194
	AI-P3	315/315	274/274	194/194	228/228	182/182	193/193	364/364	200/200	186/186

*P1–P12 eggs were laid by females housed without males and not inseminated, AI-P eggs were laid by inseminated females; – Indicates failed loci; missing data represent loci that could not be determined by genotype reconstruction.

Two of 20 inseminated females laid eggs with embryos that developed via automictic parthenogenesis, confirmed by homozygosity at all microsatellite loci. The first female that reproduced via parthenogenesis (ID 854) laid a pair of eggs four days after AI, one was infertile and the other was a parthenote. The earliest fertile eggs from AI including all females were laid 6 days after AI. Following the production of this parthenote, an additional 14 eggs were laid and seven hatched and were confirmed to be the result of AI (Fig. 4). There were 10 days between laying of the parthenote and the next AI fertilized egg. The second female that reproduced via parthenogenesis (ID 307) laid 28 eggs but only three hatched. The first egg that hatched was one of a pair of eggs laid 13 days after insemination, preceded by a pair of infertile eggs laid seven days after fertilization. This hatchling was confirmed fertilized from AI. An additional 20 infertile eggs were laid during the next 74 days followed by the last eggs laid on days 87 and 91 after AI. One egg of each of the final two pairs was infertile but the other developed via automictic parthenogenesis and hatched. There were 80 and 83 days between the AI fertilized egg and each parthenote respectively. The incidence of parthenogenesis (parthenote/total number of eggs) was 0.71% among this group of females, compared to 1.1% described above.

## Discussion

This study demonstrates that offspring can be produced from AI of whitespotted bamboo sharks with sperm cold-stored for up to 48 h. Following a single insemination, fertilized eggs were obtained for a period of 1–107 days up to 121 days after AI, confirming sperm storage for this species. Proof of paternity for whitespotted bamboo shark hatchlings was confirmed using existing and newly developed microsatellite loci, but also identified parthenotes within the same clutch of fertilized eggs for two females.

Whitespotted bamboo shark semen was able to be collected throughout the year. In the wild, these sharks reproduce seasonally with documented seasonal changes in the gonadosomatic and hepatosomatic index for both male and females^3^. However, semen collection was not attempted in these studies, so it is possible that while the gonadosomatic index of males changed seasonally, semen may have been present year-round. Additionally, there may be changes in semen quality that accompany a seasonal reproductive pattern, as has been described for seasonal terrestrial species^36^ but which were not examined in this study.

Whitespotted bamboo shark semen was confirmed to include spermatozeugmata, sperm aggregates with heads aligned and embedded in a binding matrix^21^. Spermatozeugmata are observed for many shark species^21,37^ and may serve to minimize sperm loss during copulation, increase efficiency of male and female sperm storage, or preserve longevity and motility of sperm during storage^21^. In this study a single insemination produced fertilized eggs for up to 121 days, demonstrating sperm storage for nearly 4 months. This duration of sperm storage and oviposition is similar to females newly collected from the wild in the months prior to the egg laying season and maintained in aquaria^3^ as well as females that have been maintained in aquaria for several years^4,6,12^, demonstrating the safety and efficacy of AI to facilitate reproduction of this species under managed care.

Progressively motile spermatozoa had persistent flagellar motion and rotated or rolled about their long axis as has been described for other sharks^16,22^ and rays^19^ as well as other vertebrates^38^ suggesting both motions are necessary for movement within the female reproductive tract and fertilization. Occasionally, individual progressively motile spermatozoa demonstrated periodic bursts of increased rapid forward progression especially when encountering cellular debris blocking their path forward. Spermatozoa heads were not flexible and therefore the increased forward speed may assist penetration of the protective layers surrounding the egg and be beneficial for fertilization. Spermatozoa also displayed reverse motility on occasion, similar to what has been described for other shark species^16,22^. Reverse motility is not a common spermatozoa characteristic and the role in sharks is not yet clear but could be a mechanism to liberate sperm from spermatozeugmata or be related to sperm binding sites during storage in the nidamental gland^22^. Further studies are needed to understand the significance and role of reverse motility for shark spermatozoa.

Removing the seminal plasma and resuspending in ASW resulted in greater sperm longevity of cold-stored sperm than cold storage of raw semen alone and sperm membrane integrity was maintained longer than sperm motility. These findings are in keeping with observations in the freshwater ocellate stingray *Potamotrygon motoro*, where extension of semen in saline or HBSS resulted in longer durations of motility than raw semen alone^14^. Loss of motility before PMI was observed, which was similarly observed in teleosts^39,40^. Insemination of females with raw semen cold-stored raw for 24 h or 48 h resulted in offspring production, but the low fertilization rate (7%) using 48 h semen suggests that washing sperm free of seminal plasma prior to shipping may confer some benefit to sperm survival prior to AI and should be investigated in the future.

The role of the male siphon sac in insemination supposed from mating observations is to aid in transfer of semen to the female through the clasper, presumably admixing the ejaculate with seawater, which would induce motility in the sperm. To attempt to investigate this role, ejaculates were either inseminated raw or extended 1:1 in ASW to mimic the mating process and induce sperm motility. Both inseminate preparation techniques produced a similar number of fertile eggs, suggesting uterine fluids and mixing of semen with seawater in the female’s cloaca during natural mating may activate sperm motility^16^.

Significant variation in reproductive success was observed amongst females, with some females laying few fertile eggs within a short period of time after insemination, while others produced fertilized eggs over nearly four months. Females that produced fertile eggs also laid unfertilized eggs near the end of their laying period suggesting that they may not have had sufficient viable sperm stored to fertilize all ova. This pattern could be due to improper timing of insemination. For females where AI of both uteri was bilaterally successful, the number of fertilized eggs cases laid was similar to that of naturally mated, recently collected in situ females^3^ and naturally mated aquarium females^4,5^. Of the 20 females inseminated, five did not produce any fertile eggs following insemination, including two females that were inseminated with the lowest number of sperm, possibly indicative of insufficient sperm numbers. The remaining three females received similar concentrations of sperm to fertile females inseminated with semen from the same male, though using different ejaculates, which likely precludes sperm placement issues, and might highlight gamete incompatibility and individual female fertility differences.

A wide range of fertility rates sometimes was observed for the same inseminate. This suggests that latent factors associated with the female may be more important in determining the success of a trial than inseminate characteristics, which has been shown for other species^24,41^. Females sometimes suspended egg laying for weeks or months after insemination. Similar to the unpredictable laying habits that were observed for this study, large deviations in the number of eggs laid by females in consecutive reproductive seasons has been reported for whitespotted^5^ and brownbanded bamboo females^42^*.* Timing of insemination trials is another important factor that may affect success of AI procedures. Actively ovipositing females were chosen for AI trials but in situ females mate three months before their laying season^3^. This temporal separation of a seasonal cycle for in situ sharks may be part of the reason for the observed unpredictable oviposition after AI for sharks in managed care. Zoos and aquariums manage closed populations by recommending pairings of animals that sometime require transport of individuals between institutions so the development of assisted reproductive techniques, including AI, to manage breeding is one solution that avoids risks associated with animal transfers.

An important consideration for AI studies in species that have facultative parthenogenesis as a reproductive strategy, is confirming parentage^7^. During this study, six females or 12% of the population laid one or more parthenotes during the study, two females confirmed from AI trials and minimally 4 additional females from monitoring the general population. Importantly, three parthenotes among fertilized hatchlings were confirmed in the same clutch for two females, underscoring the ability to switch reproductive modes within a very short timeframe, 10, 80 and 83 days, and in the same reproductive season. Previously the minimum time for switching between parthenogenesis and sexual development was nearly a year and between consecutive reproductive cycles for the same eagle ray female^34^. To our knowledge, this is the first occurrence of both pathenogenesis and sexual reproduction occurring within the same reproductive cycle for any fish.

Parthenogenesis is observed most often among females maintained without a male in managed care, or who are assumed to have been unsuccessful in finding a mate in the wild (sawfish). However, facultative parthenogenesis may be more prevalent than realized and occur normally in a small percentage of offspring for many shark and ray species. The realization that parthenotes may occur within the same clutch as sexually produced young, raises important questions for population managers. It should be noted that in this study females were housed without a male, and it is possible that there may be environmental drivers to initiate parthenogenesis that are not impacted by AI. Similar low percentages in background rates of parthenotes of females maintained without a male lend credence to this hypothesis. Future studies should include measuring the degree of incidence of parthenotes in populations with and without a male, to generate data that will allow more accurate population modeling. Microsatellite markers are commonly used to distinguish between individuals, establish relatedness, and prove paternity or parthenogenesis for whitespotted bamboo sharks; but because of a lack of diversity in the existing markers, possibly due to repeated pairings of the same individuals within an institution that leads to inbreeding, additional loci were required to confirm parentage^7,43^.

Results demonstrate AI combined with cold storage of semen is a practical tool for gene transfer between institutions or populations. Future research should include developing protocols for cryopreservation of shark sperm to increase the flexibility for timing of AI procedures and preserve gametes for transfer between populations worldwide. This provides a mechanism for moving genes without the need to transport sharks, removing a welfare consideration of potential transport stress. Additionally, the long-term use of this technique might be to augment in situ populations, allowing gene flow into, and out of wild populations, utilizing semen collected under short-term restraint as a sustainable resource, while eliminating concerns of long-term international transport that permanently depletes the same genes.

## Methods

### Animals and housing

Male (n = 19) and female (n = 20) whitespotted bamboo sharks (*Chiloscyllium plagiosum*) were housed at Association of Zoos & Aquariums (AZA) institutions: Adventure Aquarium, The Florida Aquarium, Aquarium of the Pacific and Ripley’s Aquarium of the Smokies. Enclosures ranged in volume from 5,500–55,000 L with seawater temperature 22–25 °C without seasonal temperature changes. Photoperiod was constant (13-15L:8-11D) except for select trials at Aquarium of the Pacific which had a natural photoperiod. Maturity of males was confirmed by the degree of clasper calcification, ability to rotate at the base, and splay open at the tip^44^. The study was carried out in compliance with the ARRIVE guidelines and was approved by the South-East Zoo Alliance for Reproduction & Conservation (SEZARC) Institutional Animal Care and Use Committee (IACUC). All experiments were performed in accordance with institutional guidelines.

### Semen collection

Semen collection was investigated by conducting and comparing two techniques- catheterization and manual expression. For semen collection by catheterization, sharks were anesthetized using 100 mg/L tricaine methanesulfonate (MS-222) (Western Chemical, Ferndale, WA, USA) buffered with 200 mg/L sodium bicarbonate. Sharks were dorsoventrally rotated, held at the water surface, and seawater drained from the cloaca. A sterile 8 Fr polyvinyl chloride catheter with two eyes and a rounded, closed tip was inserted (2–4 cm) through the urogenital papilla and slightly inclined left or right into an ampulla. Semen was extracted using gentle suction from a 1 ml or 3 ml syringe. For manual semen expression, bamboo sharks were restrained upright and out of the water, excess seawater drained from the cloaca and semen expressed using moderate bilateral ventral pressure on the region of the body immediately cranial to the pelvic girdle^11^. Semen was collected into sterile 1.5 ml conical microcentrifuge tubes and kept at ambient (~ 24 °C) temperature until processing. Semen samples were collected from individuals staggered throughout the year to encompass all months except September and December.

### Semen and sperm assessment

Semen volume was measured using a calibrated pipette. Semen osmolarity was measured using a freezing point depression method (Fiske Model 210 Micro-Osmometer, Advanced Instruments Inc., MA, USA) and pH using pH strips (ColorpHast, EMD Millipore, Billerica, MA, USA). Sperm concentration was determined using a hemocytometer after diluting semen with fresh water (1:1000) to render the sperm immotile. Motility was assessed using 3 μl raw or diluted semen (1:100 artificial seawater (ASW), 1050 mOsm, Sigma S9883, St Louis, Mo. USA). Total motility (%) was defined as the number of moving sperm and progressive motility (%) was assessed as any sperm moving with forward progression. Status was assessed on a 0–5 scale with 0 for no movement and 5 for very rapid linear progression^45^. Sperm morphology was assessed using phase contrast microscopy by examining one hundred sperm per ejaculate at 100 × oil immersion and reported as a percentage.

Sperm plasma membrane integrity (PMI), previously validated for use with elasmobranchs^22^, was assessed by incubating ASW diluted sperm suspensions in the dark for 10 min with 200 nM SYBR-14 and 24 µM propidium iodide (LIVE/DEAD Sperm Viability Kit L-7011, Molecular Probes, Inc., Eugene, Oregon, USA) and counting 100 cells using an epifluorescence microscope (Olympus B-Max 60) with filter cube U-M51005 for dual wavelength excitation. Spermatozoa fluorescing green over the head region were assessed as plasma membrane intact, and sperm fluorescing partially red or red over the head region were assessed as plasma membrane damaged^46^.

Acrosome presence was investigated using fluorescein isothiocyanate conjugated *Arachis hypogaea* agglutinin (PNA-FITC). Semen smears were made using 20 μl of semen diluted 1:100 with ASW evenly spread onto an alcohol-cleaned glass slide and allowed to air-dry. After drying, the slide was flooded with 200 μl PNA-FITC (100 μg/ml) and incubated in a dark humidified incubation chamber for 15 min at room temperature. The smear was rinsed to remove excess stain and a wet-mount examined using an epifluorescence microscope (Olympus B-Max 60) with filter cube U-M51005.

### Confocal and scanning electron microscopy

An aliquot of raw semen was preserved 1:10 in 0.1 M Sorenson’s phosphate buffer supplemented with 0.02% CaCl_2_, 0.35 M sucrose, 3.2% paraformaldehyde, and 2.5% glutaraldehyde for microscopy. Preserved sperm was stained for PMI, as described above, and imaged using a laser-scanning, confocal microscope (Zeiss 710, Thornwood, NY, USA) coupled with a Zeiss Axiophot inverted microscope in line scanning mode with a C-Apochromat 40 × Korr M27 (NA 1.2) water immersion objective, and ZEN software (Zeiss, Jena, Germany). Imaging was performed using differential interference contrast and fluorescence to highlight the boundary between the head and midpiece. Fluorescence was accomplished with laser excitation at 488 nm and 561 nm and emission collected between 500–550 nm and 575–610 nm. The length of the acrosome, head, midpiece and flagellum for 10 sperm per shark from five sharks were measured from digital photomicrographs using Fiji^47^. For scanning electron microscopy, preserved semen was dehydrated in increasing concentrations of ethanol and critical point dried (Tousimis Autosamdri-815B, Rockville, MD). Samples were mounted onto aluminum stubs, sputter coated with Au–Pd (Leica EM ACE600, Wetzlar, Germany) and examined using a Hitachi S-4700 scanning electron microscope (SEM).

### Short-term semen storage

Cold storage of whitespotted bamboo shark sperm was investigated using fresh ejaculates from four sharks with total motility of ≥ 75%. Two medias were investigated: artificial seawater (ASW), a simple osmotically balanced salt solution and Hank’s balanced salt solution (E-HBSS; 5 mM CaCl_2_, 3 mM MgCl_2_, 6 mM KCl, 0.281 M NaCl, 1 mM NaH_2_PO_4_, 8 mM NaHCO_3_, 6 mM glucose, 0.1 mM trimethylamine N-oxide (TMAO), 0.35 M urea, 0.5 mM Na_2_SO_4_, pH 7), an osmotically balanced salt solution with an energy substrate (glucose), to determine if a supplemented medium would be more supportive of cold-stored sperm longevity. Ejaculates were divided into three aliquots and stored raw or extended 1:5 in ASW or after washing to remove seminal plasma (diluted 1:10, centrifuged at 800 × *g* for 3 min and the sperm pellet resuspended 1:5 in ASW or E-HBSS). Aliquots were stored at 4 °C and evaluated as described previously for total motility and PMI on days 1, 2, 3, 5, 7, 12 and 18.

### Artificial insemination

Whitespotted bamboo sharks (n = 20) housed without a male for 2 years were selected for artificial insemination (AI). Females were housed individually in 500–1500 L habitats to monitor egg laying and embryo development six weeks before insemination. In preparation for AI, females were anesthetized using Propofol 2.5 mg/kg intraveneously or MS-222 50–75 mg/L immersion. Once anesthetized, the female was held in dorsal recumbency with her head and gills submerged and semen transferred using a sterile, semi-rigid 14 cm 3.5 Fr polypropylene catheter inserted 8–10 cm through the cloaca and diverted laterally into an oviduct, delivering half of the inseminate to each side of the paired reproductive tract. If an egg case was present in the oviduct, semen was placed cranial to the egg case by gently manipulating the catheter around the egg case or manually extracting the egg case before insemination.

Habitats were checked daily for egg cases and hatchlings. Egg cases were tagged with the date of oviposition. Egg cases that did not contain a yolk (referred to as ‘wind cases’^48^) were discarded and not included in analyses. Eggs were candled to confirm fertility by observation of a moving embryo. Egg cases collected before AI also were monitored for potential embryo development but were not included in data analyses. Oviposition was monitored for 70–216 days after insemination.

Females were inseminated with freshly collected or cold-stored semen that was raw or diluted 1:1 with seawater, at concentrations of 3–750 × 10^6^ total sperm (Supplemental Table 1). Cold-stored semen transferred between institutions was shipped overnight at 4 °C in a stryofoam box, containing a cold pack insulated from direct contact with the semen sample. For AI procedures, samples were equilibrated to room temperature prior to insemination. Motility for all inseminates was ≥ 90% with the exception of one 24-h and one 48-h cold-stored sample (Supplemental Table 1). Fertility was calculated as the percentage of eggs that developed an embryo, and hatchability as the percentage of eggs that hatched out of the total number of egg cases laid. Hatchability and fertility were adjusted to reflect hatchlings derived only from AI by excluding confirmed parthenotes (see below). Fertility duration was calculated as the number of days each female laid fertile eggs.

### Parthenogenesis

To determine the general incidence of parthenogenesis in whitespotted bamboo sharks, all eggs laid during 105 consecutive days were collected from a habitat housing 48 mature females but no males. Eggs were incubated and monitored for development as described previously. The frequency of parthenogenesis was calculated as the ratio of the number of confirmed parthenotes (described below) to the total number of eggs (excluding wind cases) and the total number of fertile eggs. Microsatellite data from the embryos and hatchlings was used to estimate the minimum number of parthenote-laying females by reconstructing maternal genotypes. For one sample (P8) the offspring was consistent with a parthenote and it was included in analyses as a parthenote, however, missing loci prevented confirmation.

### Paternity

Whole blood or a fin clip from the trailing edge of the first or second dorsal fin was collected from dams, sires, embryos and hatchlings and stored in DMSO buffer (20% DMSO, 250 mM EDTA, saturated NaCl, pH 7.5). Paternity following AI was confirmed using techniques and four microsatellite loci previously developed for this species^7,43^, together with five new species-specific microsatellite loci developed for this study (Cpl930, Cpl962, Cpl1141, Cpl1161, and Cpl1163) following existing methodology^49^. Sequences for new loci have been deposited into Genbank (Accession numbers MT237441-MT237445). Polymerase chain reaction (PCR) conditions for previously developed loci follow Ding et al*.* (2009) and Feldheim et al*.* (2010). PCRs for new loci were performed in 10 μl volumes with 1 × PCR buffer (10 mM Tris–HCl, 50 mM KCl, pH 8.3), 10 × BSA, 1.0 MgCl_2_ (Cpl1161), 1.5 mM MgCl_2_ (Cpl930 and Cpl1141), or 2.5 mM MgCl_2_ (Cpl962 and Cpl1163), 0.12 mM of each dNTP, 0.04 μM forward primer tagged with an M13 sequence on the 5′ end^50^, 0.16 μM of both the reverse primer and a fluorescently labeled universal M13 primer, and 1 U Taq polymerase. Thermal cycling began with an initial denaturation step of 94 °C for four minutes was followed by 30 cycles of 94 °C for 15 s, 58 °C for 15 s, and 72 °C for 45 s, followed by eight cycles of 94 °C for 15 s, 53 °C for 15 s, and 72 °C for 45 s. A final elongation step of 72 °C for 10 min concluded each PCR. For locus Cpl1161, a touchdown PCR was performed as follows: an initial denaturation step of 94 °C for four minutes followed by 16 cycles of 94 °C for 15 s, 68 °C for 15 s (decreasing 0.5 °C every cycle), and 72 °C for 45 s, followed by 20 cycles of 94 °C for 15 s, 60 °C for 15 s, and 72 °C for 45 s, and a final elongation of 72 °C for 10 min. PCR products (0.5 µl each) and 1.0 µl of an internal ladder (ALEXA-725)^51^ were combined with 8.5 µl HiDi Formamide and run on an ABI 3730 DNA Analyzer (Thermo Fisher Scientific). Genotypes were scored using Geneious v. v.10.0.3 (http://www.geneious.com)^52^. Paternity was determined by matching microsatellite alleles between the putative sire and dam to 88 of 112 developing embryos or hatchlings. Homozygosity for maternal alleles at all microsatellite loci coupled with a lack of paternal alleles was used to identify parthenotes^7^.

Descriptive statistics were calculated for semen and sperm parameters. Changes in PMI and motility during cold storage were examined with linear mixed effects models fit by restricted maximum likelihood with day, storage method (raw, E-HBSS or ASW) and their interaction modeled sequentially as fixed factors and repeated measures collected from the same fish accounted for by including shark as a random factor in models. Model selection was based on significance of a likelihood ratio tests between models fit by maximum likelihood that differed in fixed effects only. Model parsimony was maximized using Bayesian Information Criterion. Model residuals were examined using plots for normality, non-linearity, homoscedasticity and outliers. Pearson correlation was used to determine the relationship between motility and PMI for cold-stored semen samples. Differences in fertility for AI trials between ejaculates split and inseminated raw and diluted were evaluated using a paired t-test. Statistical analyses were conducted using R Version 4.0.0^53^ with ggplot2 Version 3.3.0^54^, lme4 Version 1.1–23 and a critical probability level of 0.05.

## Supplementary Information

Supplementary Information.

## Data Availability

The datasets generated during and/or analysed during the current study are included in this published article (and its Supplementary Information files) or available from the corresponding author on reasonable request. Sequences for new whitespotted bamboo microsatellite loci have been deposited into Genbank, Accession numbers MT237441-MT237445.
