# Supplementary material for: Artificial insemination and parthenogenesis in the whitespotted bamboo shark Chiloscyllium plagiosum
Source: Sci Rep. 2021 May 13;11:9966. doi: 10.1038/s41598-021-88568-y (PMC8116330; doi:10.1038/s41598-021-88568-y)
Supplement: Supplementary file 1 — Supplementary Information. [file 41598_2021_88568_MOESM1_ESM.pdf]

Artificial insemination and parthenogenesis in the whitespotted bamboo shark *Chiloscyllium plagiosum*

Jennifer T. Wyffels, Lance M. Adams, Frank Bulman, Ari Fustukjian, Michael W. Hyatt, Kevin A. Feldheim and Linda M. Penfold

Supplemental Table 1. Inseminate characteristics and outcomes of whitespotted bamboo shark *Chiloscyllium plagiosum* artificial insemination trials

Female ID	Male ID-Ejaculate	Total Motility	Inseminate Age	Semen Treatment	Sperm Number (million)	Wind Egg Cases	Egg Cases Before First Fertile	Fertile Egg Cases	Embryonic Death
854	320-E1	≥90%	fresh	extended	100	2	2	11	1
101	381-E2	≥90%	fresh	extended	750	0	0	17	2
283	381-E4	≥90%	fresh	extended	100	0	3	20	2
268	381-E5	≥90%	fresh	raw	100	0	0	21	7
793	395-E1	≥90%	24 hours^	raw	100	4	1	12	0
036	320-E3	≥90%	fresh	raw	300	2	2	12	1
581	381-E1	≥90%	fresh	raw	750	1	0	4	1
512	381-E3	≥90%	fresh	raw	100	1	1	3	0
034	381-E3	≥90%	fresh	raw	100	0	1	5	0
370	2969-E1	10%	24 hours*	raw	750	0	4	2	0
785	381-E4	≥90%	fresh	raw	100	2	1	1	0
376	381-E1	75%	48 hours	raw	750	0	10	1	0
307	320-E1	≥90%	fresh	extended	100	0	0	3	0
626	395-E1	≥90%	24 hours^	extended	100	14	3	1	0
583	395-E1	≥90%	24 hours^	extended	100	11	7	1	0
063	381-E2	≥90%	fresh	raw	750	0	-	-	-
775	381-E3	≥90%	fresh	raw	100	0	-	-	-
551	381-E5	≥90%	fresh	extended	100	2	-	-	-
065	320-E2	≥90%	fresh	extended	3.0	5	-	-	-
095	320-E2	≥90%	fresh	extended	3.0	1	-	-	-

* semen shipped overnight from Adventure Aquarium, NJ, to Aquarium of the Pacific, CA

^ semen shipped overnight from The Florida Aquarium, FL, to Ripley's Aquarium of the Smokies, TN
